# Intra-abdominal multi-organ involvement of kaposiform hemangioendothelioma in a child with Kasabach–Merritt phenomenon and contrast-enhanced ultrasound findings: a case report

**DOI:** 10.3389/fmed.2025.1705267

**Published:** 2025-12-08

**Authors:** Shu Luo, Anjie Chen, Jiaojiao Zhou, Yi Ji, Shuguang Jin, Juxian Liu

**Affiliations:** 1 Division of Ultrasound, West China Hospital of Sichuan University, Chengdu, Sichuan, China; 2 Division of Pediatric Surgery, West China Hospital of Sichuan University, Chengdu, Sichuan, China

**Keywords:** Kaposiform hemangioendothelioma, Kasabach-Merritt phenomenon, contrast-enhanced ultrasound, pancreatic neoplasms, child

## Abstract

**Background:**

Kaposiform hemangioendothelioma (KHE) is a rare, locally aggressive vascular tumor that occurs in infancy and early childhood and is associated with significant morbidity. A severe complication of KEH is Kasabach–Merritt phenomenon (KMP), a life-threatening consumptive coagulopathy accompanied by thrombocytopenia.

**Case presentation:**

We report a rare case of KHE complicated by KMP, with multifocal involvement of the pancreatic head, liver, biliary tract, and duodenal papilla. Contrast-enhanced ultrasound (CEUS) revealed characteristic imaging features of the pancreatic head lesion, demonstrating inhomogeneous hypoenhancement during the arterial and portal phases, followed by complete washout and absence of enhancement in the late phase.

**Conclusion:**

After the failure of multiple therapeutic interventions, the patient successfully underwent a pancreatoduodenectomy. This case underscores that surgical resection should be considered for KHE when it is feasible and safe. Furthermore, recognizing the distinctive CEUS features of pancreatic KHE is crucial for accurate diagnosis and avoiding diagnostic pitfalls.

## Background

Kaposiform hemangioendothelioma (KHE) is a rare, endothelium-derived, locally aggressive vascular tumor that typically occurs during infancy or early childhood. KHE usually has a cutaneous origin, affecting the extremities, cervicofacial region, and torso body wall; it can also involve certain abdominal organs. The abnormal proliferation of capillaries and lymphatic vessels can activate platelets in the blood, leading to Kasabach–Merritt phenomenon (KMP), which is characterized by intractable thrombocytopenia, hypofibrinogenemia, coagulation dysfunction, and severe anemia (1, 2). KHE imaging usually includes magnetic resonance imaging (MRI), computed tomography (CT), gray-scale ultrasound, Doppler ultrasound, and ultrasonic elasticity. However, to date, the use of contrast-enhanced ultrasound (CEUS) has not been reported, to the best of our knowledge. The present study describes a rare case of KHE in the pancreatic head, which invaded multiple abdominal organs and was accompanied by KMP and multiple secondary lesions, resulting in a complex and challenging diagnosis. CEUS revealed that the KHE lesions in the pancreatic head exhibited an enhancement pattern typical of a malignant tumor. The patient was treated with various medical therapies, followed by surgical resection of the lesions, leading to recovery.

## Case presentation

**Figure 1:**
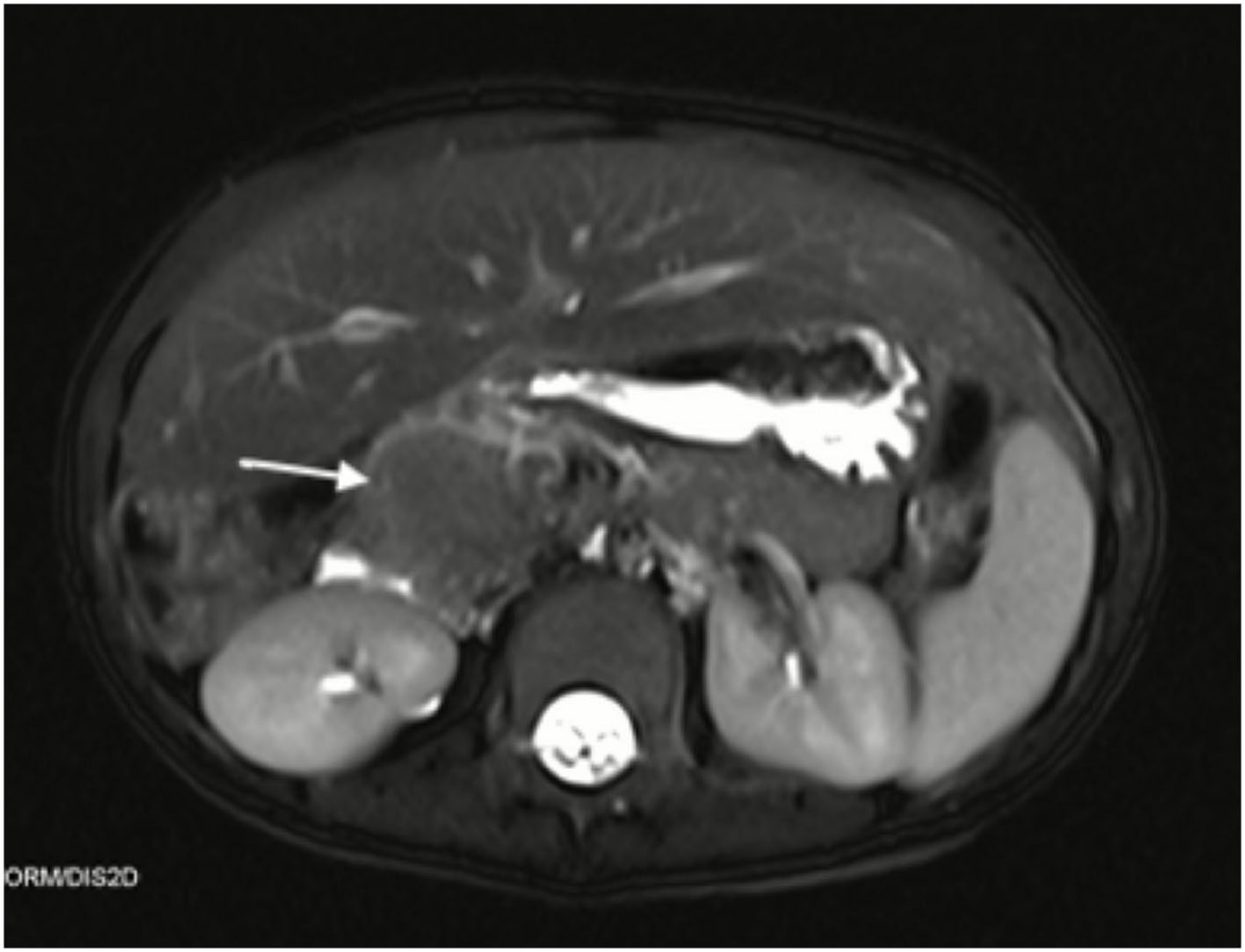
Axial T2-weighted and contrast-enhanced MRI of the abdomen demonstrating a mildly hypointense mass with inhomogeneous delayed enhancement.

The patient, a 2-year-old girl, was suddenly found to have unusual ecchymosis on her feet. A pediatrician found that the platelet count of the child had decreased significantly, and the lowest platelet value was 4 × 10^9^/L. The initial clinical diagnosis was primary immune thrombocytopenia, and the child was treated with gamma globulin and prednisone. When the child was 4 years old, she developed unexplained abdominal pain and the levels of liver enzymes suddenly increased. Enhanced MRI demonstrated an abnormal focus in the pancreatic head, measuring ~2.3 × 1.3 cm. The T2-weighted image revealed that the tumor showed a mild hypointense signal, and enhanced MRI showed inhomogeneous delayed enhancement of the mass (Figure 1). MRI demonstrated the invasive nature of the lesion, which appeared as a mass with extensive pancreatic involvement, extending to abut the right liver.

**Figure 2:**
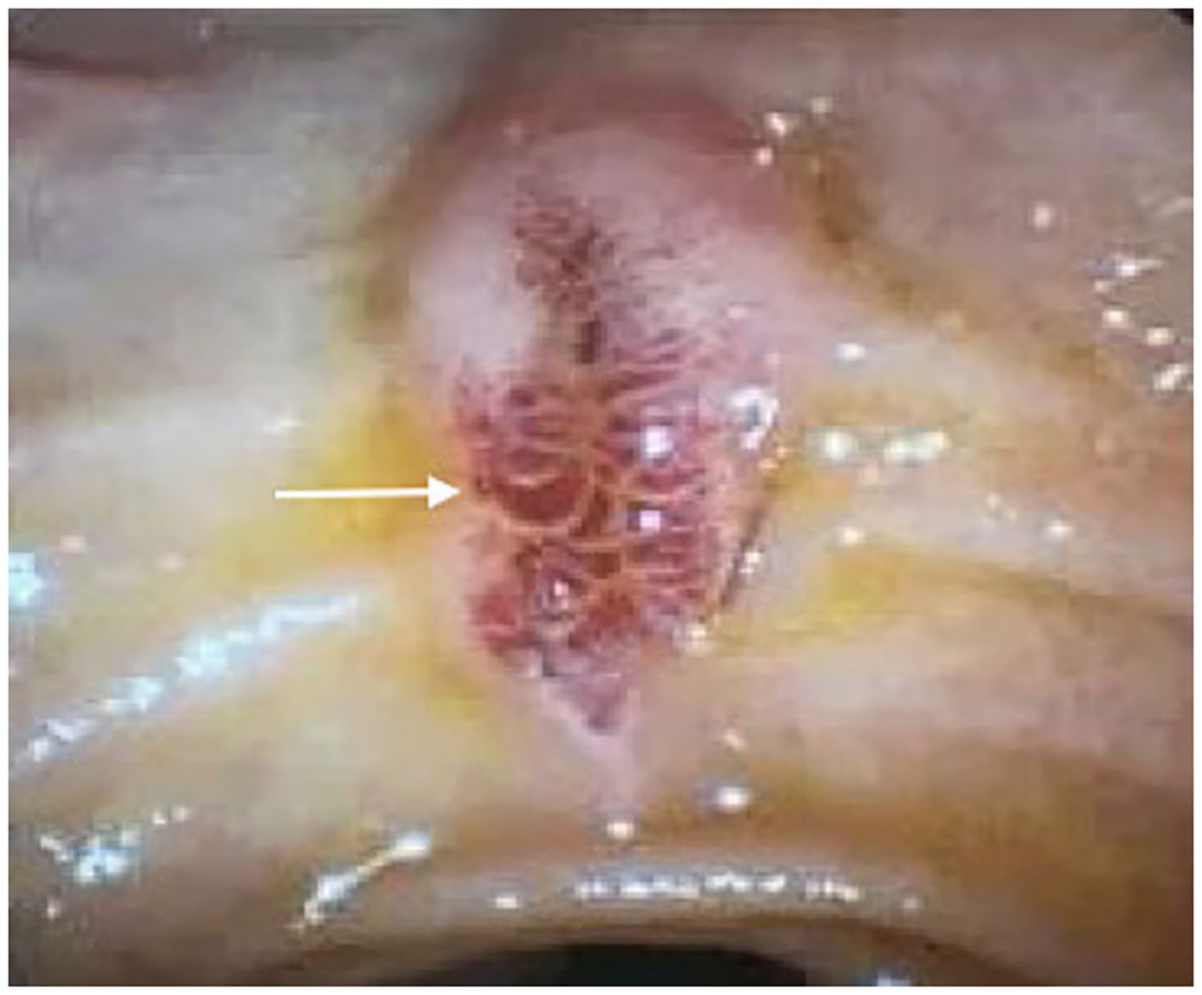
Endoscopic retrograde cholangiopancreatography showing a series of raspberry-like vascular malformations in the duodenal papilla.

Liver biopsy ruled out common autoimmune liver diseases, viral hepatitis, and metabolic liver disease. Endoscopic retrograde cholangiopancreatography (ERCP) images revealed (Figure 2) a series of raspberry-like vascular malformations in the duodenal papilla, along with an extensive lesion in the pancreatic head beneath this plexus. Pathological examination identified only a cavernous hemangioma, with no evidence of other tumor cells.

**Figure 3:**
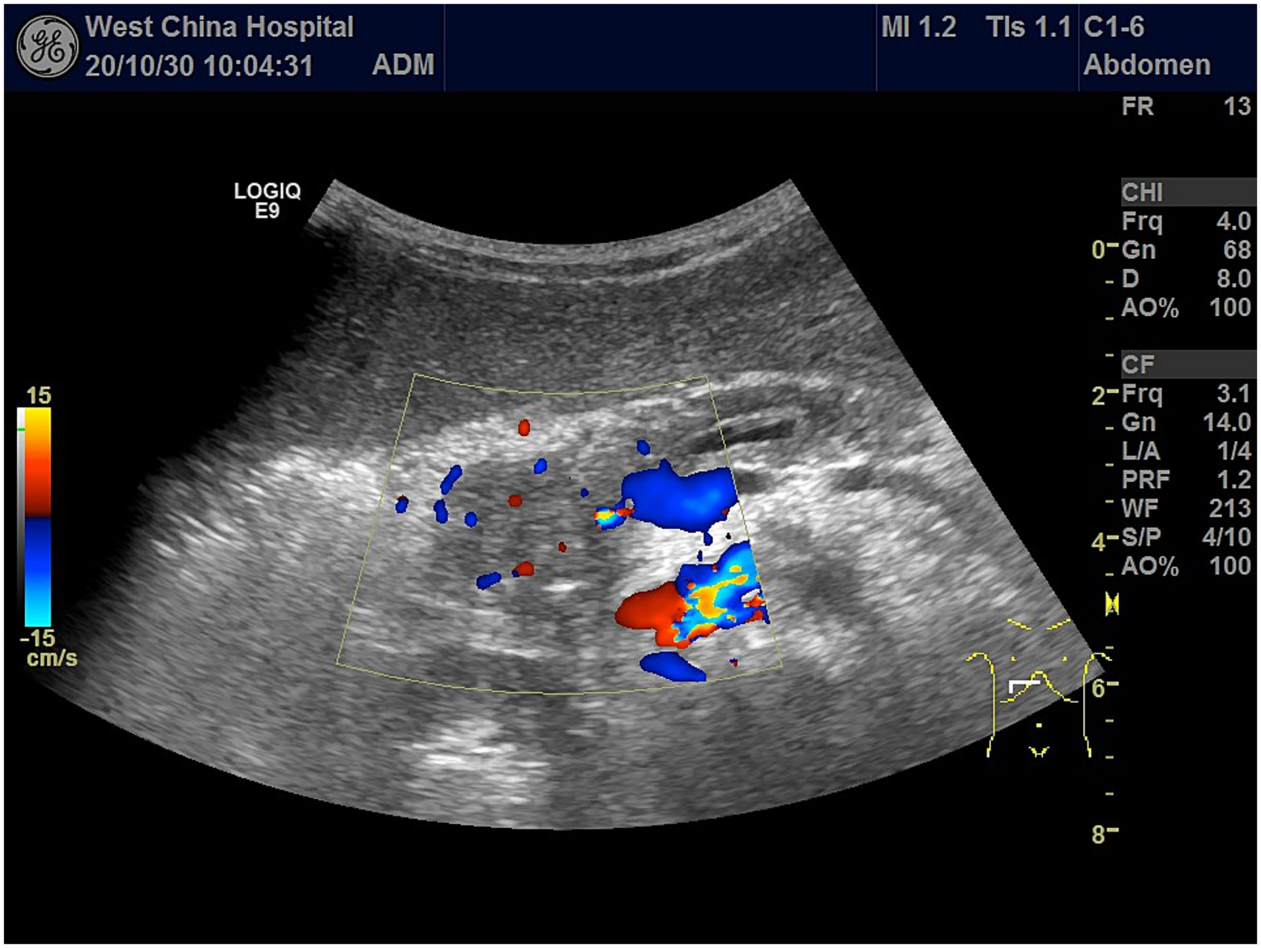
Color Doppler flow imaging of the pancreatic head showing an irregular hypoechoic mass with ill-defined margins, measuring approximately 4.0 × 2.9 × 3.5 cm, with punctate internal vascularity (Adler Grade 2).

**Figure 4:**
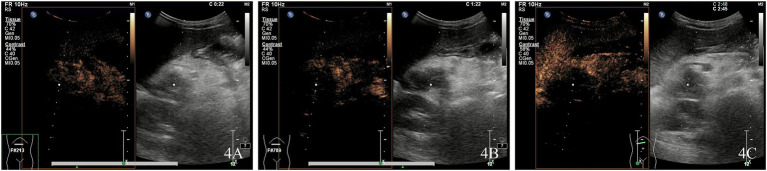
Kaposiform hemangioendothelioma of the pancreatic head demonstrating rapid, heterogeneous hypoenhancement at 22 s post-contrast injection, with persistent heterogeneous hypoenhancement in the arterial phase **(A)** and progressively reduced enhancement in the portal venous phase **(B)**. The lesion exhibited early washout starting at 80 s, with near-complete washout in the delayed phase **(C)**.

Ultrasound examination revealed a hypoechoic mass in the pancreatic head, measuring ~4.0 × 2.9 × 3.5 cm, with unclear borders and irregular morphology. Punctate blood flow signals (Adler blood flow grade 2) were observed within the lesions (Figure 3). Enhancement of the pancreatic parenchyma was observed 19 s after contrast injection. The lesion exhibited rapid, inhomogeneous hypoenhancement at 22 s after contrast administration and consistently maintained this inhomogeneous hypoenhancement. The imaging findings showed a hypoenhanced lesion in the arterial phase (Figure 4A) and lower enhancement in the portal venous phase (Figure 4B). By 80 s, the lesion began to washout, with nearly complete washout observed in the late phase (Figure 4C). This suggested the presence of a tumorigenic lesion. Gray-scale ultrasound revealed that the right side of the liver was significantly reduced in size compared to the left side of the liver. High-frequency ultrasound revealed findings consistent with liver cirrhosis, including an unsmooth liver envelope and heterogeneous parenchymal echogenicity; this was supported by a markedly elevated liver stiffness measurement of 26.1 kPa on shear wave elastography. The flow velocity in the portal venous system was markedly reduced. The gallbladder wall was thickened (0.5 cm), and the spleen was enlarged (13.4 × 3.4 cm). These ultrasound findings indicated that the patient may have cirrhosis and portal hypertension.

The characteristic CEUS findings suggested the presence of neoplastic lesions rather than inflammatory areas. Following discussion among clinicians, an extended pancreaticoduodenectomy was performed. During surgery, clinicians confirmed the presence of a cancerous lesion in the pancreatic head. Samples from the pancreatic tumor, pancreatic parenchyma, and adjacent liver and bile ducts were obtained. Pathological analysis ultimately confirmed that the child had a rare case of KHE with multi-organ involvement, including the pancreas, liver, biliary tract, and duodenal papilla. Immunohistochemical staining of the pancreatoduodenal arcade was performed. The pathological results confirmed the diagnosis of KHE complicated by KMP. Following surgery, the child received aspirin treatment, and the platelet level increased to 250 × 10^9^/L. During the follow-up period, there was no recurrence of skin ecchymosis or pancreatitis, and the platelet count remained within the normal range. The repeated ultrasound examination revealed no tumor recurrence. The pancreatic condition and cirrhotic changes in the liver showed marked improvement.

## Discussion

In the present case report, the onset of the disease was occult, with skin ecchymosis and thrombocytopenia as the primary clinical manifestations, followed by recurrent pancreatitis. In this case report, KHE involved multiple organs, including the pancreatic head, liver, biliary tract, and duodenal papilla, resulting in complex clinical symptoms and imaging manifestations. Color Doppler flow imaging revealed blood flow signals in the lesions of the pancreatic head. The characteristics of KHE included inhomogeneous hypoenhancement in the arterial and portal phases, with no enhancement in the late phase. The lesion demonstrated poor enhancement across all dynamic phases on CEUS examination. These findings were consistent with the characteristics of pancreatic malignancies, such as ductal adenocarcinoma. However, sch tumors are extremely rare in children (3, 4). Based on the disease history, laboratory findings, and imaging features, we diagnosed pancreatic KHE combined with KMP.

## Conclusion

In light of these findings, imaging information was provided to the clinician. A mass was identified in the pancreatic head, probably malignant, which was then surgically resected. Following treatment, the patient, who initially failed to respond to other treatments, showed rapid recovery after surgery. Certainly, the CEUS characteristics of KHE need to be further investigated and analyzed.

## Data Availability

The original contributions presented in the study are included in the article/supplementary material, further inquiries can be directed to the corresponding author.
